# Performance Evolution and Degradation Mechanism of Chemically Bonded Phosphate Ceramic Cement Under Freeze–Thaw Cycles

**DOI:** 10.3390/ma18235298

**Published:** 2025-11-24

**Authors:** Bo Pang, Runqing Liu, Yuanquan Yang, Yunpeng Cui

**Affiliations:** 1 School of Civil Engineering, Liuzhou Institute of Technology, Liuzhou 545616, China; pangbo0829@sylu.edu.cn; 2 School of Materials Science and Engineering, Shenyang Ligong University, Shenyang 110159, China

**Keywords:** chemical bonded phosphate ceramics cement, freeze–thaw cycle, MgKPO_4_·6H_2_O, Potassium magnesium phosphate hexahydrate, hydration product

## Abstract

This study investigates the performance variations in chemically bonded phosphate ceramic (CBPC) cement under different media (water and 3% NaCl solution) environments subjected to varying numbers of freeze–thaw cycles, including changes in compressive strength, mass loss rate, phase composition, microstructure, external pH, and ion concentration, with the aim of elucidating its long-term durability degradation mechanisms and microstructural evolution. The results show that both the mass and compressive strength of CBPC cement first increase and then decrease with increasing freeze–thaw cycles. After 400 cycles, the compressive strength decreases by 29.91% in water and 25.16% in salt solution. The pH value rises with cycling, along with increased concentrations of K^+^, Mg^2+^, and PO_4_^3−^, while Na^+^ and Cl^−^ concentrations decrease in salt solution. XRD/Rietveld analysis reveals that the content of MgKPO_4_·6H_2_O decreases from 28.1% to 19.5% (water) and 20.7% (salt), with a gradual reduction in crystallinity. TG/DTG and FTIR results confirm these findings, showing extensive microcracking in hydration products, which aligns with the observed macro-performance changes.

## 1. Introduction

Magnesium phosphate cement (MPC) is a cementitious material formed through the reaction between magnesium oxide (MgO) and phosphate. Compared to traditional Portland cement, MPC offers advantages such as rapid hardening [1,2,3,4], high early strength [5,6], low shrinkage, and excellent bonding properties [7,8]. It has found extensive applications in rapid repair [9], solidification/stabilization [10,11,12], thermal insulation materials [13], and biomedical engineering [14]. Silica fume (SF), a powdery material with extremely fine particles typically ranging from 0.1 to 1 micrometer in diameter, is a by-product of ferrosilicon or silicon metal production. It primarily consists of amorphous SiO_2_ and exhibits a high specific surface area and pozzolanic activity [15,16,17]. Due to its unique physical and chemical properties, silica fume is widely used in cement-based materials [18] to enhance their performance. Previous studies [15,19,20,21,22] have demonstrated that incorporating silica fume as an admixture into Portland cement significantly improves its compressive strength, resistance to chloride ion penetration and sulfate attack, and freeze–thaw cycling. Furthermore, the amorphous silica in silica fume, owing to its more disordered structure and lower bond energy of Si-O bonds, exhibits high reactivity [23,24]. Under the alkaline environment (typically with a pH of 10–12) generated during MPC hydration, the surface Si-O bonds of SiO_2_ are promoted to break, accelerating the gradual dissolution of amorphous SiO_2_ to produce soluble silicic acid. This soluble silicic acid reacts with Mg^2+^ in MPC hydration products to form magnesium silicate hydrate (M-S-H) gel, thereby enhancing MPC performance [25,26]. Although, existing research has preliminarily explored the effects of silica fume incorporation on the compressive strength, water resistance, and setting time of MPC-based materials [19,27,28,29]; for instance, Ahmet Pehlivan [30] found that adding silica fume to basalt fiber-reinforced MPC significantly improved its mechanical properties, and Bo Pang [31] reported that MgO-SiO_2_-KH_2_PO_4_ cement exhibited excellent corrosion resistance in sulfate environments, primarily due to its unique product formation and dense structure. Cong Ma [32] found that SF plays a role in nucleation and filling during the hydration process of MPC, which accelerates its hydration process and improves the density of the matrix, and during the reaction process, amorphous magnesium silicate (M-S-H) is generated. However, there is still a serious lack of systematic research on the durability degradation mechanism, performance evolution law, and microstructure damage characteristics of the SF-modified MPC system under freeze–thaw cycles. Therefore, this study aims to fill this gap by systematically investigating the macro-performance and micro-mechanism of SF-modified MPC under both water and salt freeze–thaw conditions. The findings are expected to provide crucial data support for the reliable application of this material in extreme environmental civil engineering.

Therefore, this study employs SF to modify MPC and investigates the effects of different freeze–thaw environments (water freeze–thaw and salt freeze–thaw) and varying numbers of freeze–thaw cycles on the modified cement. This research aims to systematically investigate the macro-performance and micro-mechanism of SF-modified MPC under freeze–thaw conditions, thereby providing data support for its application in cold environments.

## 2. Experiment

### 2.1. Materials

**Figure 1:**
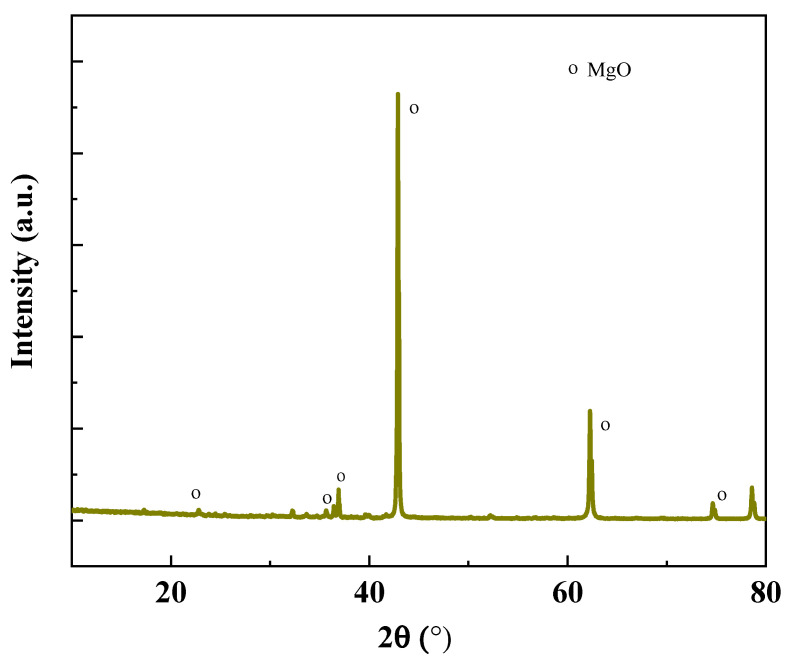
XRD pattern of the MgO.

**Figure 2:**
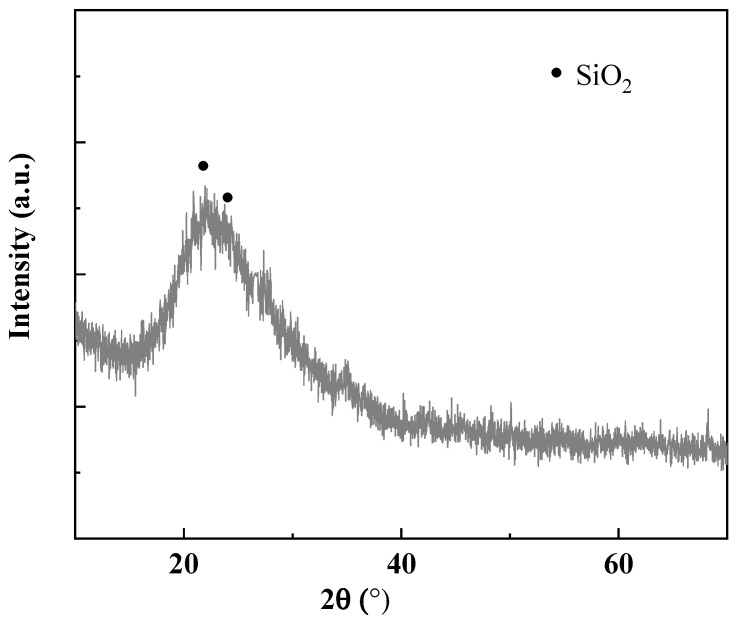
XRD pattern of the SF.

**Figure 3:**
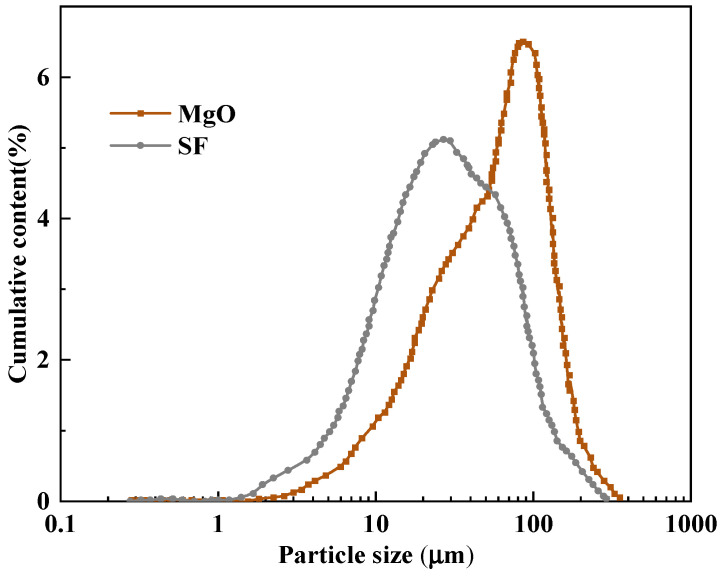
The particle size distribution of the MgO and SF.

In this investigation, magnesium oxide (MgO) sourced from Liaoning, China, was employed at temperatures ranging from 1500 to 1700 °C. Silica fume (SF), also obtained from Liaoning, China, was utilized, with its primary chemical component being amorphous silicon dioxide. The chemical compositions of both MgO and SF, as determined by X-ray Fluorescence (XRF) analysis, are presented in Table 1 and Table 2, respectively. The mineral composition of MgO and SF determined by XRD are shown in Figure 1 and Figure 2. The particle size distributions of MgO and SF were determined by a laser particle size analyzer, as shown in Figure 3.

The raw materials used in this study were as follows: industrial-grade potassium dihydrogen phosphate (KH_2_PO_4_) and borax (Na_2_B_4_O_7_·10H_2_O), each with a purity of >99%, and sodium chloride (NaCl, >99% purity) of analytical grade, procured from Sinopharm Chemical Reagent Factory. The mixing water for the CBPC cement pastes was conventional tap water, the quality of which was in accordance with the requirements of the Chinese standard JGJ 63-2006 (“Standard for Water for Concrete”) [33].

### 2.2. Sample Preparation

The CBPC cement was formulated with MgO, SF, and KH_2_PO_4_ as its main components. The mix design, specified in Table 3, was optimized for enhanced mechanical properties and workability by adopting an M/P molar ratio of 4, a borax content equivalent to 5% of dead-burned magnesia, and a W/B mass ratio of 0.18. Various amounts of SF were added as a replacement for MgO. The preparation procedure involved an initial dry blending of the solid constituents (MgO, KH_2_PO_4_, borax, and SF) in a beaker at 50 rpm for 30 s. After adding the measured mixing water, the paste was mixed at low speed (50 rpm) for another 30 s and then at high speed (100 rpm) for 1 min. All mixing was conducted at ambient temperature (20 ± 2 °C). The resulting slurry was cast into a 20 mm × 20 mm × 20 mm mold and demolded after 1 h. The demolded specimens were air-cured for 24 d under controlled conditions (20 ± 2 °C, 50% humidity), followed by a 4 d water immersion to achieve saturation. Then, initial compressive strength and quality testing were conducted, followed by rapid freeze–thaw testing (water freezing, 3% NaCl freezing). The number of low-temperature freeze–thaw cycles in this study was set to 50, 100, 200, and 400. The temperature regime for a single cycle was set to cool down from 20 °C to −30 °C for 1.5 h, with a cooling stage at −30 °C for 1 h, heating stage from −30 °C to 20 °C for 1.5 h, and constant-temperature stage at 20 °C for 1 h, for a total of 5 h. After the freeze–thaw cycle test of CBPC cement, the water surrounding the sample was taken for ICP and pH testing.

### 2.3. Experimental Test

The performance degradation and microstructural evolution of the CBPC cement specimens after exposure to freeze–thaw cycles were evaluated through a series of macro-performance tests and microstructural analyses. These included the mass loss rate, compressive strength, pH value, and ion concentration of the surrounding solution, conducting X-ray diffraction (XRD), Fourier-transform infrared spectroscopy (FTIR), thermogravimetric analysis (TG/DTG), and scanning electron microscopy (SEM). The detailed procedures for each test are described below.

#### 2.3.1. Mass Loss Rate

The mass of the specimen, designated as m_0_, was measured prior to testing. Subsequent to being subjected to n cycles of freezing and thawing in different solutions, the mass was re-measured and recorded as *m*. The mass loss rate was then defined by Formula (1):

(1) $$∆m=\frac{m-m_{0}}{m_{0}}\;*\;100\%$$

where

Δ*m* is the mass loss rate;
*m*_0_ is the initial mass;
*m* is the mass after the nth cycle.

#### 2.3.2. Compressive Strength

Samples with different soaking ages were tested to failure on the universal testing machine under a continuous load of 2.4 kN/s. The test data were recorded upon completion of this process.

#### 2.3.3. pH

Upon completion of the freeze–thaw cycling tests, the solution surrounding the specimens was collected. The pH value of this solution was then measured using a Mettler Toledo S400-k pH meter (Columbus, OH, USA).

#### 2.3.4. TG/DTG

In this experiment, the TG/DSC analyzer (Netzsch STA 449 F3, Selb, Germany) was used for detection. A sample with a mass of about 10 mg was weighed and placed in an alumina crucible. The temperature was raised from 20 °C to 700 °C at a heating rate of 10 °C/min.

#### 2.3.5. XRD

X-ray diffraction analysis was conducted using an X-ray diffractometer (Ultima IV, Rigaku, Japan). Analysis was performed using Cu-Kα radiation with a wavelength of 1.541874 Å at 40 mA and 40 kV. The 2θ scanning range was set from 10° to 50°, with a scanning speed of 2°/min in continuous scanning mode.

#### 2.3.6. FTIR

An FTIR spectrometer (Thermo Fisher Nicolet iS50, Madison, WI, USA) was used to mix 1 mg test powder samples with potassium bromide (KBr) at a 1:100 ratio that were pressed into transparent sheets for spectral testing.

#### 2.3.7. SEM

Scanning electron microscopy (S-3400N, Hitachi, Ltd., Tokyo, Japan) was performed with the following specific parameters: a voltage of 230 V, a frequency of 50/60 Hz, a current of 8A, and an accelerating voltage of approximately 20 kV.

## 3. Results and Discussion

### 3.1. Mass Loss Rate

**Figure 4:**
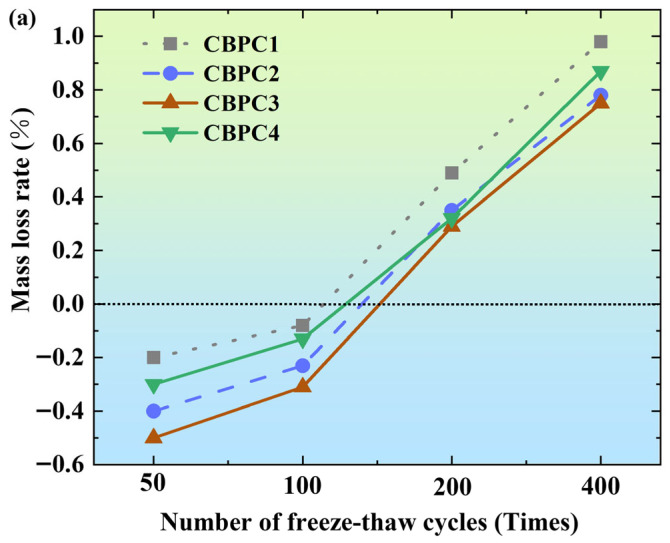

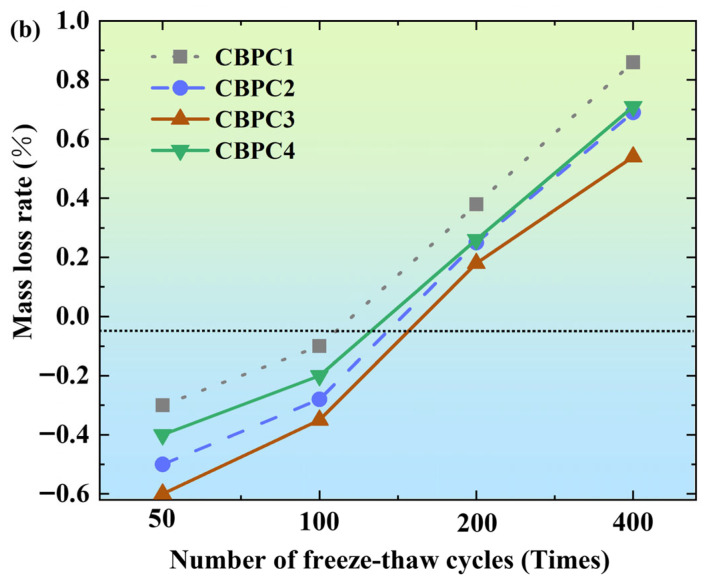
Mass loss rate of samples while soaking in solutions ((**a**): water freezing and thawing; (**b**): NaCl freezing and thawing).

As shown in Figure 4a, after different numbers of freeze–thaw cycles in a water medium, the mass of CBPC cement exhibits a trend of initially increasing and then decreasing. At 50 freeze–thaw cycles, the mass change rates of specimens with different mix proportions are −0.2% (CBPC1), −0.4% (CBPC2), −0.5% (CBPC3), and −0.3% (CBPC4), respectively, indicating that CBPC cement shows a net mass gain phenomenon in the early stage of freeze–thaw cycles. When the number of cycles increases to 100, although the mass change rates show an upward trend (CBPC1: −0.08%; CBPC2: −0.23%; CBPC3: −0.31%; CBPC4: −0.13%), they still remain negative. It is speculated that in the short-term freeze–thaw cycles, water infiltrates into the pores of CBPC cement and freezes, leading to an increase in mass, and the short-term freeze–thaw cycles do not cause damage to the cement. Among them, when the silica fume content is 15%, the mass loss is the smallest. It is speculated that in the short-term freeze–thaw cycles, different amounts of silica fume result in different quantities of expansive hydration products formed in the CBPC cement, leading to an increase in specimen mass. When the number of freeze–thaw cycles increases to 200, the mass loss rate begins to turn positive, indicating that the mass starts to decrease at this point. When the number of freeze–thaw cycles reaches 400, with a silica fume content of 15%, the mass loss is the smallest at 0.75%, indicating that an appropriate amount of silica fume enhances the freeze–thaw resistance of CBPC cement in water. The mass loss during the freeze–thaw process is mainly due to the fact that as the number of cycles increases, the ice formation pressure generated by the freezing of water in the pores causes severe damage to the pore walls.

As shown in Figure 4b, after different numbers of freeze–thaw cycles in a 3% NaCl solution, the overall trend is the same as that in the water freeze–thaw cycles. At 50 and 100 freeze–thaw cycles, the mass losses are negative. At 200 and 400 freeze–thaw cycles, the mass loss values are positive. Under different numbers of freeze–thaw cycles, the MSPPC3 with a silica fume content of 15% consistently shows the best performance in terms of mass loss rate. At 400 freeze–thaw cycles, its mass loss rate is 0.54%, indicating that an appropriate amount of silica fume enhances the salt freeze–thaw resistance of CBPC cement. In addition, in the NaCl solution, NaCl can lower the freezing point of water, so the mass loss is relatively smaller compared to that in the water medium freeze–thaw cycles.

### 3.2. Compressive Strength

**Figure 5:**
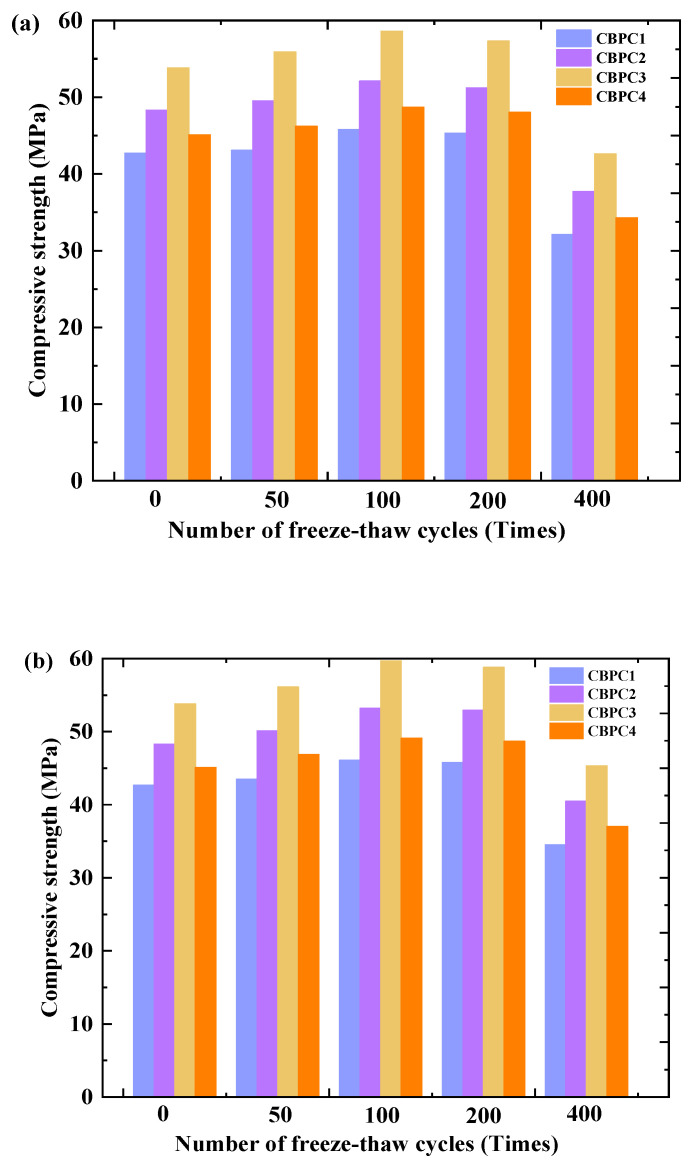
Compressive strength of samples with different freeze–thaw environments and cycle times ((**a**): water freezing and thawing; (**b**): NaCl freezing and thawing).

In the freeze–thaw cycles within the water-frozen environment depicted in Figure 5a, the compressive strength of CBPC cement exhibits a trend of initially increasing and then decreasing. At 100 freeze–thaw cycles, the compressive strength reaches its peak values, which are 45.8 MPa, 52.1 MPa, 58.6 MPa, and 48.7 MPa, representing an increase of 7.26–8.92% compared to the compressive strength without freeze–thaw treatment. At 200 freeze–thaw cycles, the compressive strength of CBPC cement begins to decline, with a decrease range of 1.09–2.22%. At 400 freeze–thaw cycles, the compressive strength of CBPC cement experiences a significant drop, reaching 32.1 MPa, 37.7 MPa, 42.6 MPa, and 34.3 MPa, which are decreases of 29.91%, 27.64%, 27.30%, and 29.57%, respectively, compared to the peak values. In Figure 5b, after undergoing freeze–thaw cycles in a 3% NaCl solution, the compressive strength of CBPC cement follows a similar trend of initially increasing and then decreasing, consistent with the changes observed in the water-frozen environment. At 100 freeze–thaw cycles, the compressive strength reaches its peak values of 46.1 MPa, 53.2 MPa, 59.7 MPa, and 49.1 MPa, respectively, after which the compressive strength decreases with an increase in the number of cycles. At 200 freeze–thaw cycles, the decreases in compressive strength compared to the peak values range from 0.56% to 1.51%. At 400 freeze–thaw cycles, the compressive strength of CBPC cement shows a substantial decline, reaching 34.5 MPa, 40.5 MPa, 45.3 MPa, and 37 MPa, respectively, which are decreases of 25.16%, 23.87%, 24.12%, and 24.64% compared to the peak values. It is speculated that before 100 freeze–thaw cycles, the hydration of CBPC cement is still ongoing, continuously contributing to its strength. After 100 freeze–thaw cycles, the hydration gradually ceases, and the damaging effect of freeze–thaw cycles on CBPC cement begins to manifest. In addition, by comparing freeze–thaw cycles in water and salt environments, it was found that the compressive strength of samples subjected to freeze–thaw cycles in NaCl solution is higher than that of the samples subjected to freeze–thaw cycles in water. It is speculated that this is mainly because the crystallization produced by the NaCl solution accumulates within the hydration products of CBPC cement, and the accumulated amount has not yet reached a level that would generate expansive pressure in the products, thus exerting a positive influence.

### 3.3. pH and Ion Concentration Analysis

**Figure 6:**
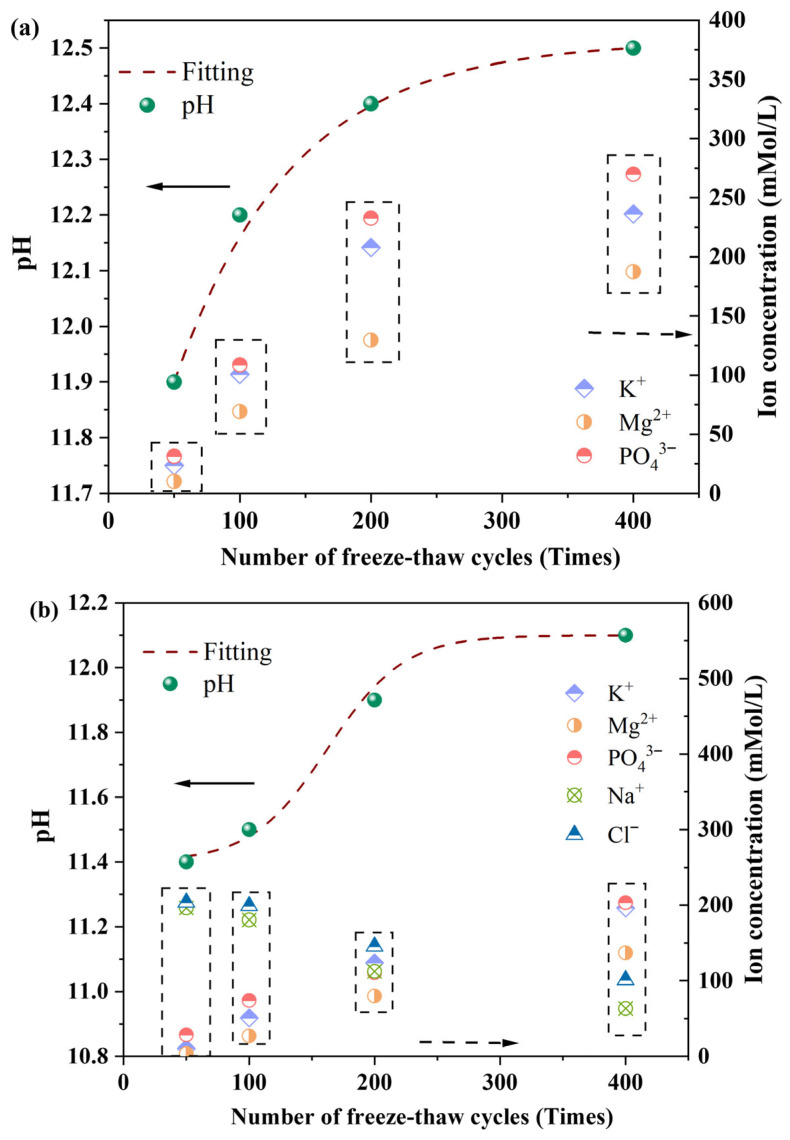
pH and ion concentration analysis of samples with different freeze–thaw environments and cycle times ((**a**): water freezing and thawing; (**b**): NaCl freezing and thawing).

As shown in Figure 6a, the pH value of the surrounding water and the results of Inductively Coupled Plasma (ICP) tests on the CBPC cement after water freeze–thaw cycling treatment indicate that with an increase in the number of freeze–thaw cycles, the surface water pH value exhibits an increasing trend toward alkalinity. Simultaneously, the ion concentrations of K^+^, Mg^2+^, and PO_4_^3−^ all show an increasing trend. This phenomenon can be attributed to the cumulative damage effect of the specimens caused by freeze–thaw cycling, which leads to an aggravated degree of internal hydration product destruction. Driven by the concentration gradient, the ion concentrations of K^+^, Mg^2+^, and PO_4_^3−^ in the surface water gradually increase.

As illustrated in Figure 6b, the pH value of the surface water and the ICP test results of the CBPC cement after freeze–thaw cycling treatment in a 3% NaCl solution reveal that the surface water pH value also shows an increasing trend toward alkalinity, and the ion concentrations of K^+^, Mg^2+^, and PO_4_^3−^ also exhibit an increasing trend. In contrast, the ion concentrations of Na^+^ and Cl^−^ show a decreasing trend. This difference suggests that in a salt freeze–thaw environment, Na^+^ and Cl^−^ gradually diffuse into the internal pore network of the CBPC cement matrix under the action of an external concentration gradient, resulting in a gradual decrease in the Na^+^ and Cl^−^ concentrations in the surface layer. Meanwhile, the continuous destruction of internal hydration products still maintains the increasing trend of K^+^, Mg^2+^, and PO_4_^3−^ leaching.

Therefore, the changing trends in pH value and ion concentration detection demonstrate that both water freeze–thaw and salt freeze–thaw cycles, to a certain extent, can cause irreversible damage and erosion to the hydration products of CBPC cement.

### 3.4. FTIR Analysis

**Figure 7:**
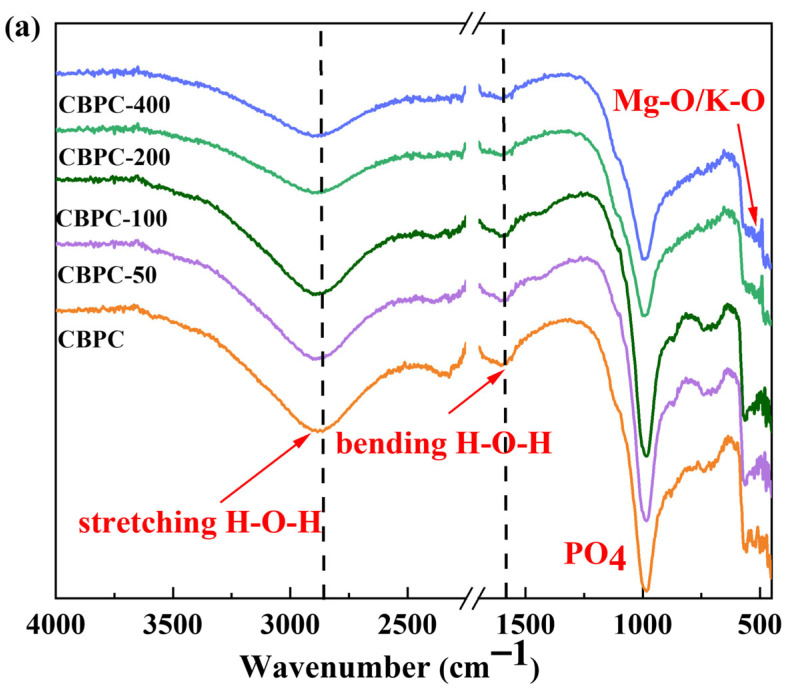

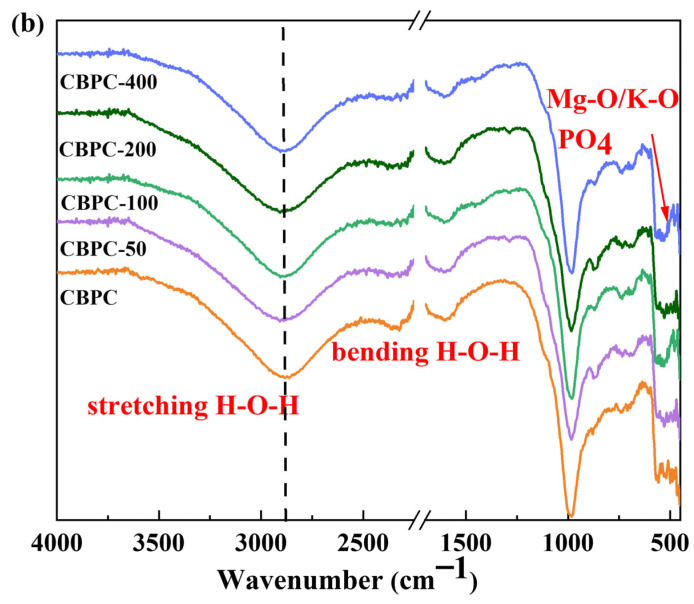
FTIR of samples with different freeze–thaw environments and cycle times ((**a**): water freezing and thawing; (**b**): NaCl freezing and thawing).

Figure 7a presents the infrared spectra of CBPC cement subjected to freeze–thaw cycles under different environments. As can be observed from the CBPC in the figure, the original peak profile exhibits a broad H-O-H absorption band within the wavenumber range of 2500 cm^−1^–3000 cm^−1^ and another H-O-H absorption band in the range of 1400 cm^−1^–1900 cm^−1^, indicating the existing forms of water during the freeze–thaw cycling process. When the freeze–thaw cycling occurs within a water environment for less than 100 cycles, the H-O-H absorption band within the wavenumber range of 2500 cm^−1^–300 cm^−1^ remains unchanged. However, after more than 200 freeze–thaw cycles, this H-O-H absorption band within the 2500 cm^−1^–3000 cm^−1^ range shifts toward higher wavenumbers. These changes suggest a decrease in the role of water molecules. The H-O-H absorption band within the 1400 cm^−1^–1900 cm^−1^ range follows the same pattern, indicating that the water in the hydration products is affected. The vibration of PO_4_ shifts toward higher wavenumbers with an increasing number of freeze–thaw cycles, suggesting that substances containing PO_4_ in the products are influenced.

In Figure 7b, during freeze–thaw cycling in NaCl solution, the absorption bands of H-O-H, PO_4_, and Mg-O show almost no changes or only insignificant variations, indicating that the impact of freeze–thaw cycling in a NaCl solution on CBPC cement is less significant than that in a water environment. This is consistent with the results of the changes in mechanical properties.

### 3.5. XRD Analysis

**Figure 8:**
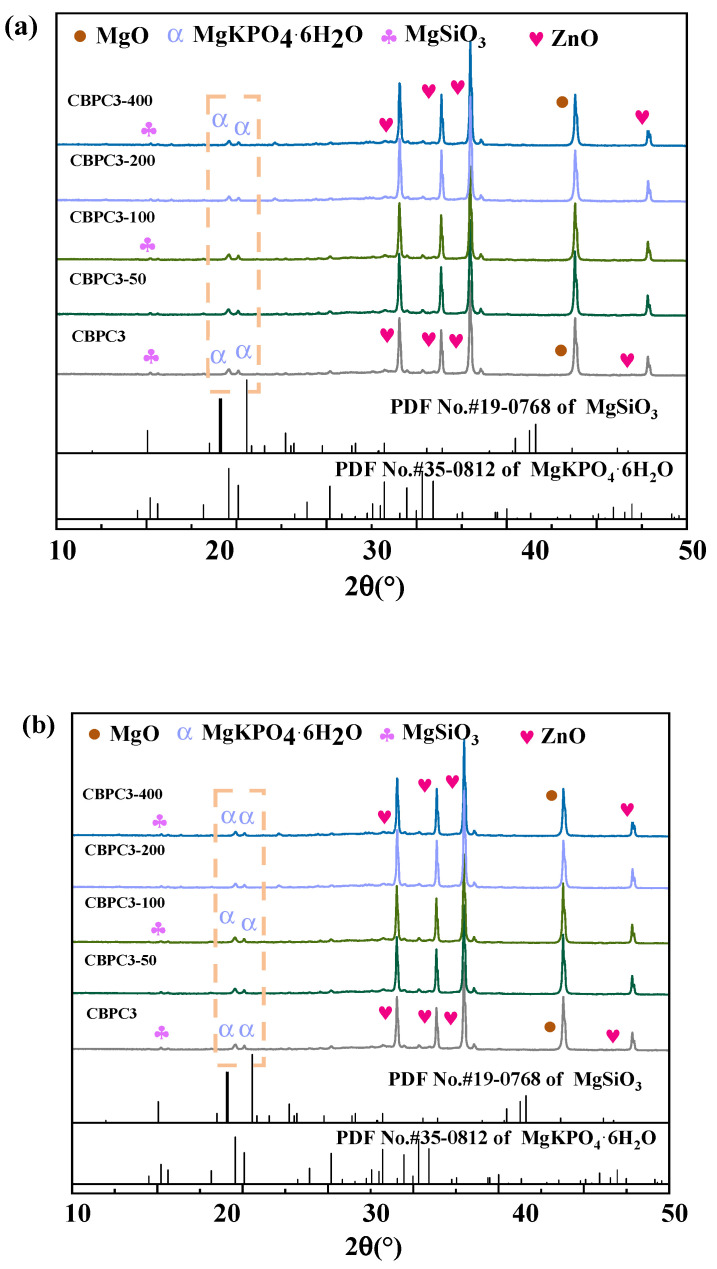
XRD patterns of samples with different freeze–thaw environments and cycle times ((**a**): water freezing and thawing; (**b**): NaCl freezing and thawing).

**Figure 9:**
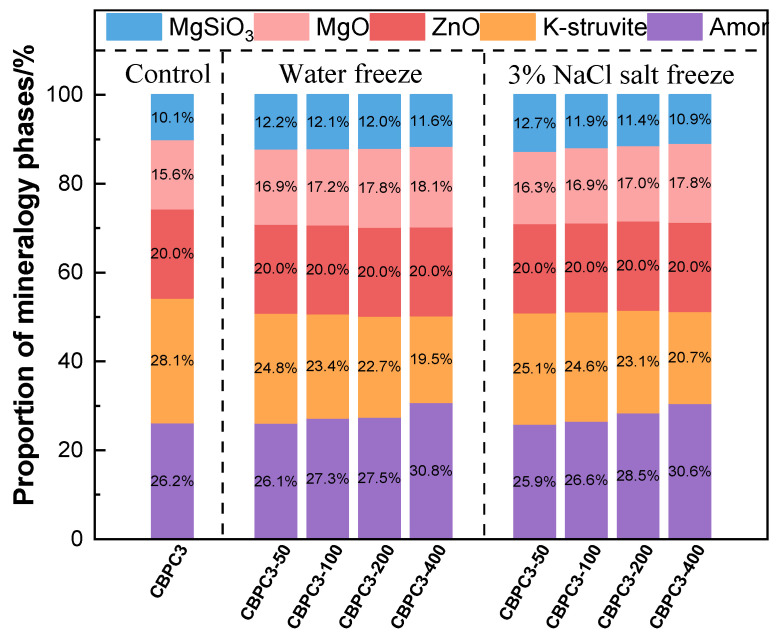
XRD/Rietveld quantitative analysis of samples with different freeze–thaw environments and cycle times.

The XRD patterns of CBPC cement subjected to freeze–thaw cycling in water are presented in Figure 8a. The main diffraction peaks correspond to MgKPO_4_·6H_2_O, MgSiO_3_, unreacted MgO, and the quantitative calibration standard, ZnO. Among them, MgKPO_4_·6H_2_O and MgSiO_3_ are the primary contributors to the compressive strength of CBPC cement. The phase types of CBPC cement remain unchanged after different numbers of freeze–thaw cycles in water; instead, variations are mainly observed in the crystallinity of certain products and their content. According to the quantitative analysis results from XRD/Rietveld in Figure 9, as the number of freeze–thaw cycles increases, the content of MgKPO_4_·6H_2_O decreases sequentially. Specifically, it gradually drops from 28.1% in the non-freeze–thaw-cycled sample to 19.5% after 400 freeze–thaw cycles. In contrast, the content of MgSiO_3_ exhibits a different trend. The MgSiO_3_ content in all freeze–thaw-cycled samples is higher than that in the non-freeze–thaw-cycled sample. The MgSiO_3_ content is 10.1% in the non-freeze–thaw-cycled sample and decreases from 12.2% after 50 freeze–thaw cycles to 11.6% after 400 freeze–thaw cycles. Meanwhile, the content of the amorphous phase increases synchronously with the number of freeze–thaw cycles, gradually rising from 26.2% in the non-freeze–thaw-cycled sample to 30.8% after 400 freeze–thaw cycles.

The XRD patterns of CBPC cement after different numbers of freeze–thaw cycles in a 3% NaCl solution are shown in Figure 8b. The phases are the same as those in the water freeze–thaw cycling, mainly consisting of MgKPO_4_·6H_2_O, MgSiO_3_, diffraction peaks of unreacted MgO, and the quantitative calibration standard ZnO, with no change in phase types. According to the quantitative analysis results from XRD/Rietveld in Figure 9, the changes in thmain hydration products, MgKPO_4_·6H_2_O and MgSiO_3_, are similar to those in the water freeze–thaw environment. The content of MgKPO_4_·6H_2_O gradually decreases from 28.1% in the non-freeze–thaw-cycled sample to 20.7% after 400 freeze–thaw cycles. The MgSiO_3_ content in the freeze–thaw-cycled samples is still higher than that in the non-freeze–thaw-cycled sample, and after freeze–thaw cycling, it gradually decreases from 12.7% after 50 freeze–thaw cycles to 10.9% after 400 freeze–thaw cycles.

The XRD peak structures indicate that with an increasing number of freeze–thaw cycles, the content and crystallinity of MgKPO_4_·6H_2_O gradually decrease. Similarly, the content of MgSiO_3_ also decreases due to the increase in freeze–thaw cycles, but its content in the water freeze–thaw cycling environment is higher than that in the salt freeze–thaw cycling environment. Combined with the changes in compressive strength, it is evident that MgKPO_4_·6H_2_O and MgSiO_3_ are the main strength-contributing phases, with MgKPO_4_·6H_2_O contributing more to the strength than MgSiO_3_.

### 3.6. TG/DTG Analysis

**Figure 10:**
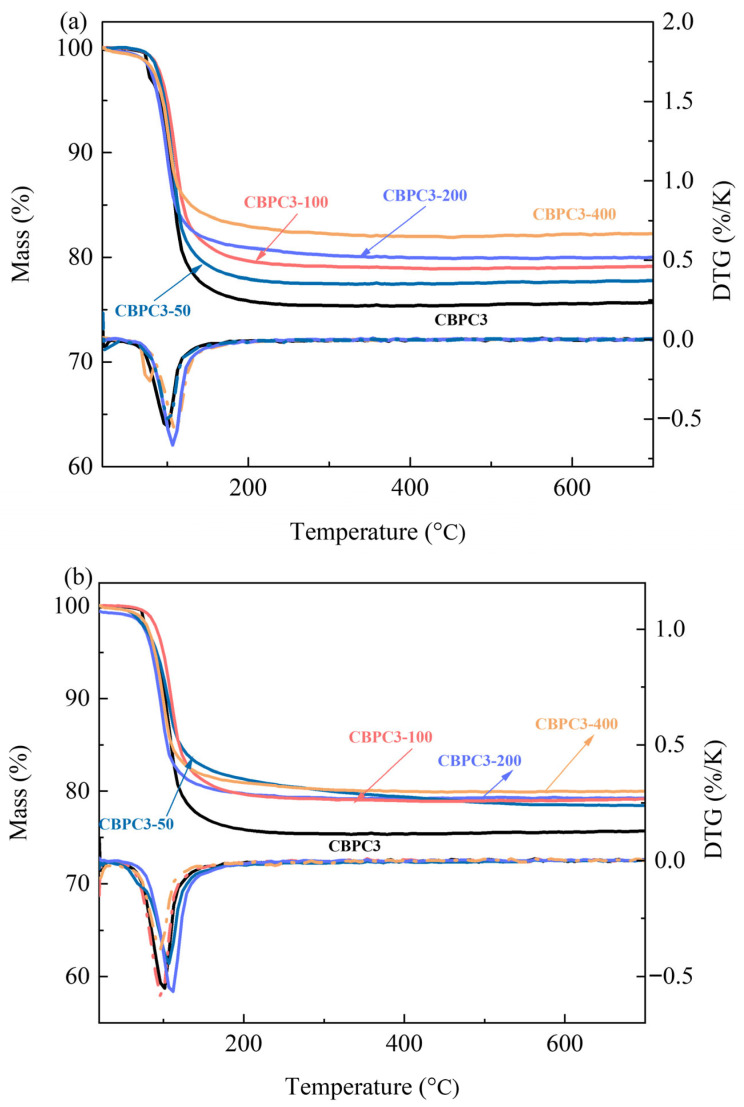

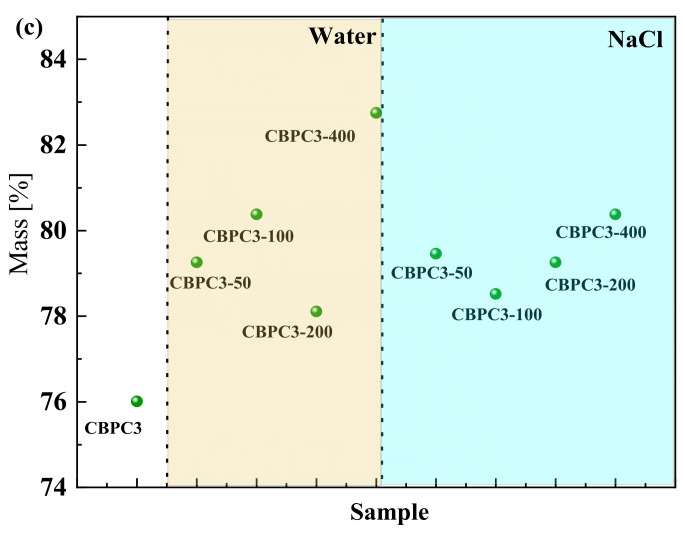
TG/DTG analysis of samples with different freeze–thaw environments and cycle times ((**a**): water freezing and thawing; (**b**): NaCl freezing and thawing; (**c**): mass loss value).

The results of thermogravimetric (TG)/derivative thermogravimetric (DTG) analysis reveal that, after water freeze–thaw cycling treatment, as shown in Figure 10a, all the CBPC cement specimens with different proportions exhibit a prominent weight loss peak around 100 °C. The mass loss process initiates at approximately 50 °C, with a significant increase in the weight loss rate at 70 °C, reaching its peak near 110 °C. This weight loss characteristic is highly consistent with the thermal decomposition behavior of MgKPO_4_·6H_2_O [22,23]. TG curve analysis indicates that the specimens without water freeze–thaw cycling exhibit a significantly higher weight loss rate, while the specimens subjected to different numbers of water freeze–thaw cycles show varying weight loss rates.

As illustrated in Figure 10b, after freeze–thaw cycling treatment in a NaCl solution, the weight loss peak characteristics of all the CBPC cement specimens with different proportions are consistent with those obtained from the water freeze–thaw cycling tests, all displaying the typical decomposition characteristics of MgKPO_4_·6H_2_O. The mass loss values in the TG curves are presented in Figure 10c. The specimens without freeze–thaw cycling exhibit a significantly higher mass loss rate compared to those subjected to freeze–thaw cycling treatment. Moreover, when the number of freeze–thaw cycles reaches 400, the specimens show the highest mass loss rate.

The mass loss rates obtained from TG/DTG analysis are positively correlated with the content of MgKPO_4_·6H_2_O determined by XRD/Rietveld quantitative analysis. This is primarily because the mass loss of CBPC cement after heating is almost entirely attributed to MgKPO_4_·6H_2_O.

### 3.7. SEM Analysis

**Figure 11:**
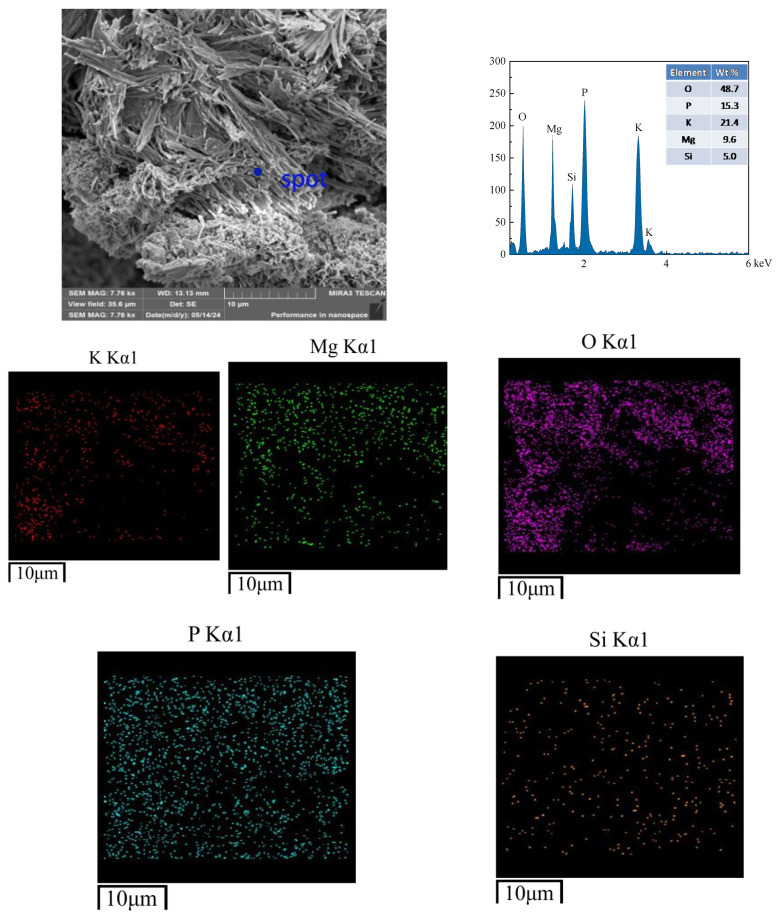
Microstructure of the samples without freezing and thawing.

**Figure 12:**
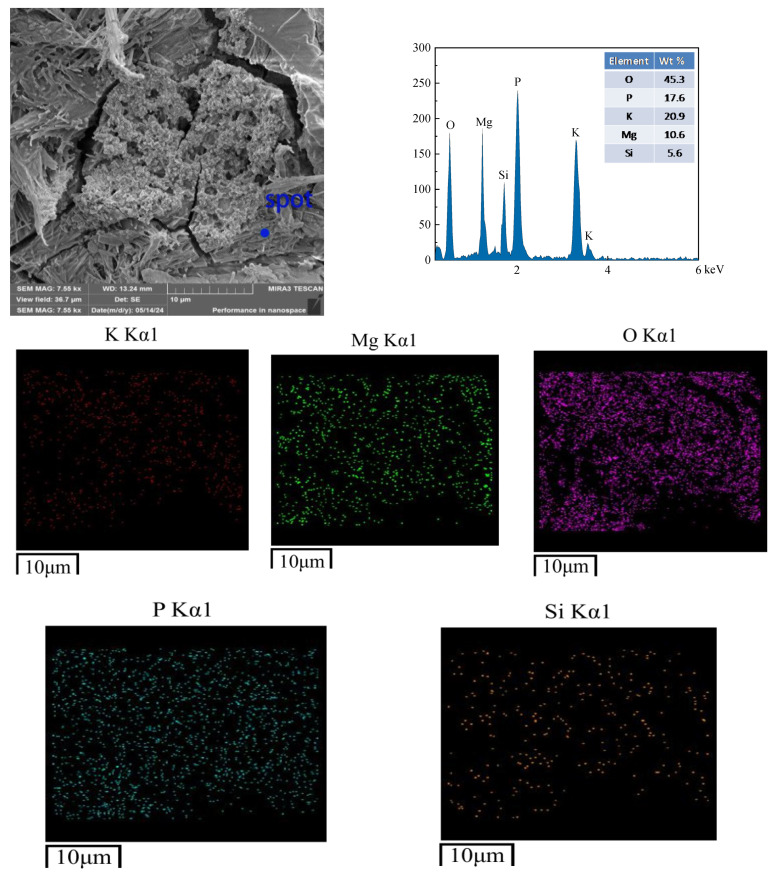
Microstructure of the samples with water freezing and thawing for 400 cycles.

**Figure 13:**
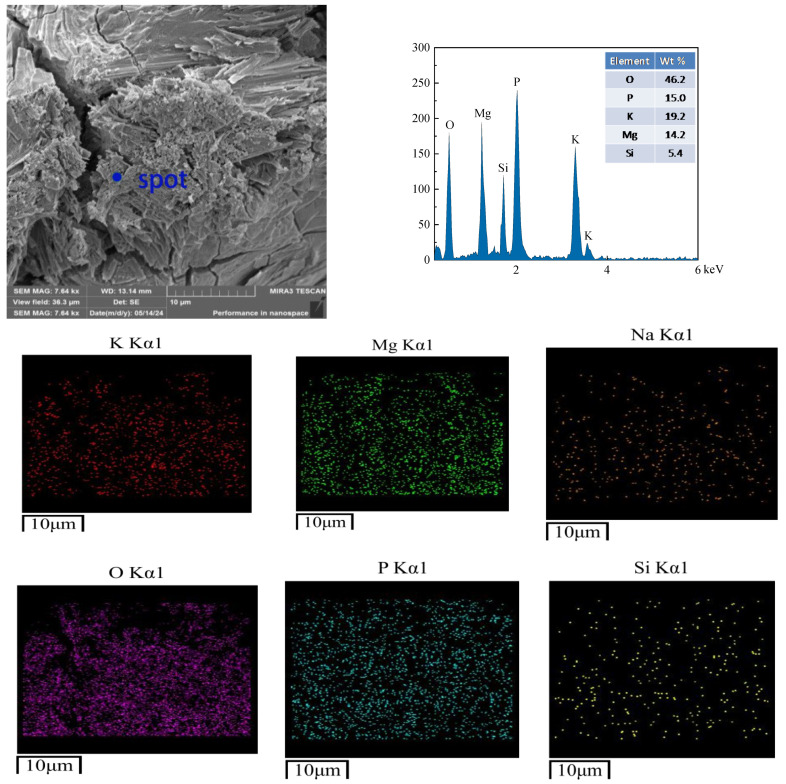
Microstructure of the samples with NaCl freezing and thawing for 400 cycles.

Microstructural analysis was conducted on samples subjected to 400 freeze–thaw cycles in water and salt (3% NaCl solution) environments. The non-freeze–thaw-cycled CBPC cement is depicted in Figure 11. The hydration products of the CBPC cement exhibit strip-like and irregular blocky shapes. EDS analysis reveals the distribution and presence of five elements, namely K, P, O, Mg, and Si. The rod-shaped crystalline product, MgKPO_4_·6H_2_O, becomes slender and tightly clustered together, encapsulating unreacted MgO and resulting in a dense structure, which is consistent with the compressive strength values. At this point, there are small cracks in the microstructure, with a maximum size of 1.07 µm, indicating that the structure is already very dense. As shown in Figure 12, in the hydration products of the CBPC cement after 400 freeze–thaw cycles in water, needle-like substances are observed to be cemented together with irregular blocks, and a significant number of cracks are present. The maximum crack width of the product under this condition is 1.92 µm and the longest is 13.44 µm. EDS analysis still detects the distribution and presence of the five elements, K, P, O, Mg, and Si. In Figure 13, for the hydration products of the CBPC cement after 400 freeze–thaw cycles in a 3% NaCl solution, EDS analysis reveals the presence of several elements, including Na, K, P, O, Mg, and Si. Although cracks are also present in the overall structure, the longest crack size is 10.57 µm and their quantity is reduced compared to those in the water freeze–thaw-cycled samples, which is consistent with the patterns observed in the mechanical properties.

## 4. Conclusions

This study investigated the performance variations in CBPC cement, including compressive strength, mass loss rate, phase composition, micro-morphology, external pH, and ion concentration changes, under different freeze–thaw cycling conditions in various media (water and 3% NaCl solution). The following conclusions were drawn:

(1) Among the tested mixes, the CBPC3 sample with a 15% silica fume content demonstrates optimal overall performance. It exhibits the lowest mass loss (0.75% in water and 0.54% in salt solution after 400 cycles) and the highest compressive strength retention under both freeze–thaw environments, indicating that this proportion most effectively enhances the microstructure density and freeze–thaw resistance of the CBPC matrix.
(2) The compressive strength results reveal that the compressive strength of CBPC cement initially increases and then decreases with an increasing number of freeze–thaw cycles in different media. The compressive strengths decrease by 29.91% and 25.16% under water freeze–thaw and salt freeze–thaw cycling, respectively. The higher compressive strength under salt freeze–thaw cycling conditions is attributed to the accumulation of NaCl crystals.
(3) The results regarding the external pH and ion concentrations of CBPC cement after freeze–thaw cycling show that the pH value increases with the number of freeze–thaw cycles under different media conditions. Simultaneously, the ion concentrations of K^+^, Mg^2+^, and PO_4_^3−^ all increase, while the ion concentrations of Na^+^ and Cl^−^ decrease during salt freeze–thaw cycling. Freeze–thaw cycling in different media causes irreversible erosion to the hydration products of CBPC cement.
(4) XRD/Rietveld quantitative analysis demonstrates that the content of MgKPO_4_·6H_2_O decreases sequentially with an increasing number of freeze–thaw cycles, while the content of MgSiO_3_ exhibits a different trend. After 400 water freeze–thaw cycles, the content of MgKPO_4_·6H_2_O decreases from 28.1% to 19.5%, whereas after 400 salt freeze–thaw cycles, it gradually decreases from 28.1% to 20.7%. Additionally, the crystallinity of MgKPO_4_·6H_2_O gradually decreases. The results from TG/DTG and FTIR analyses are consistent with the quantitative analysis results and align with the macroscopic performance variations.
(5) The microstructural results of the products reveal that freeze–thaw cycling induces numerous cracks in the hydration products of CBPC cement, resulting in an overall non-dense structure. The elements detected by EDS are consistent with those in the phase products. Furthermore, the microstructural changes are in line with the macroscopic and phase variations.
(6) Future prospects: Based on the findings of this study, future research can be directed towards the following: (i) investigating the performance of the optimal mix (15% SF) under coupled deterioration conditions, such as freeze–thaw cycles combined with mechanical loading or chemical corrosion; (ii) exploring the long-term durability (beyond 400 cycles) and field application performance in real cold environments.

## Figures and Tables

**Table 1:** Chemical composition of the MgO.

Oxide	MgO	CaO	SiO_2_	Fe_2_O_3_	Na_2_O	Al_2_O_3_	K_2_O
Content (%)	93.1	2.1	2.2	0.4	0.03	0.19	0.25

**Table 2:** Chemical composition of the SF.

Oxide	SiO_2_	CaO	Fe_2_O_3_	K_2_O	Na_2_O	SO_3_	C	Loss
Content (%)	95.6	1.3	0.7	0.45	0.2	0.55	0.6	0.6

**Table 3:** Mix proportions of CBPC cement (%).

Sample	Water/Cement Ratio	Borax (% Cement)	Mg/P Ratio	SF (% by Mass of Cement)
CBPC1	0.18	5	4	5
CBPC2				10
CBPC3				15
CBPC4				20

## Data Availability

The original contributions presented in this study are included in the article. Further inquiries can be directed to the corresponding author.
